# Psychological burden, quality of life, problems, and parental concerns of single mothers with cancer: a cross-sectional comparison

**DOI:** 10.1007/s00520-025-09225-y

**Published:** 2025-02-11

**Authors:** Steffen Holsteg, Maike K. Klett, Anna-Maria Kisić, Rebecca Horbach-Bremen, Manuela Brüne, Marc Dohmen, Barbara Drueke, Nicole Ernstmann, Franziska Geiser, Lina Heier, Christian Heuser, Andrea Icks, Jens Panse, Andrea Petermann-Meyer, Anja Viehmann, André Karger

**Affiliations:** 1 Clinical Institute of Psychosomatic Medicine and Psychotherapy, Medical Faculty and University Hospital Düsseldorf, Heinrich Heine University Düsseldorf, Düsseldorf, Germany; 2 Center for Integrated Oncology Aachen Bonn Cologne Düsseldorf (CIO ABCD), Düsseldorf, Germany; 3 Department of Hematology, Oncology, Hemostaseology and Stem Cell Transplantation, Faculty of Medicine, RWTH Aachen University, Aachen, Germany; 4 Institute for Health Services Research and Health Economics, Centre for Health and Society, Medical Faculty and University Hospital Düsseldorf, Heinrich Heine University Düsseldorf, Düsseldorf, Germany; 5 Institute for Medical Psychology and Medical Sociology, Faculty of Medicine, RWTH Aachen University, Aachen, Germany; 6 Chair of Health Services Research, Institute of Medical Sociology, Health Services Research and Rehabilitation Science, Faculty of Medicine and University Hospital Cologne, University of Cologne, Cologne, Germany; 7 Center for Health Communication and Health Services Research (CHSR), Department for Psychosomatic Medicine and Psychotherapy, University Hospital Bonn, Bonn, Germany; 8 Department of Psychosomatic Medicine and Psychotherapy, University Hospital Bonn, University of Bonn, Bonn, Germany; 9 Department of Clinical Pharmacy and Toxicology, Maastricht University Medical Center, Maastricht, The Netherlands; 10 Department of Clinical Pharmacy, Cardiovascular Research Institute Maastricht, CARIM, Maastricht University, Maastricht, The Netherlands; 11 Institute for Health Services Research and Health Economics, German Diabetes Center, Leibniz Center for Diabetes Research at Heinrich Heine University Düsseldorf, Düsseldorf, Germany

**Keywords:** Parental cancer, Single mothers, Minor children, Psycho-onocology, Distress, Parent experience

## Abstract

**Purpose:**

Single motherhood is associated with increased psychosocial risks, affecting both mothers and their minor children. However, little is known about the specific psychosocial impact of maternal cancer in single mothers (SMs). This study compared psychological burden, quality of life, specific problems, and parental concerns between SMs and partnered mothers (PMs) affected by cancer and caring for minor children.

**Methods:**

Cross-sectional analysis of baseline data from a multicenter, non-randomized, controlled trial in Germany (Family-SCOUT). SMs and PMs affected by cancer were assessed for psychological burden (anxiety, depression, distress), quality of life, practical, family, emotional, spiritual/religious, and physical problems and parental concerns.

**Results:**

A total of 54 SMs and 245 PMs were included. SMs reported more practical problems (*p* = 0.008, *d* = 0.44) and parental concerns than PMs (*p* = 0.011, *d* = 0.40). After controlling for demographic and clinical group differences, practical problems (*p* = 0.009, OR = 1.53) and parental concerns (*p* = 0.015, OR = 1.73) remained significantly associated with single motherhood. SMs and PMs did not differ in anxiety, depression, distress or quality of life. Overall, a large proportion of mothers reported clinically relevant elevated levels of anxiety (71.9%), depression (46.8%) and heightened distress (82.3%).

**Conclusion:**

In this sample, the psychological burden of mothers with cancer who care for minor children did not differ based on whether they were parenting alone or together with a co-parent. However, SMs reported more practical problems and parental concerns than PMs, emphasizing the need for targeted support in practical problem-solving and child care for SMs.

Trial Registration Number: NCT04186923, 5. December 2019.

**Supplementary Information:**

The online version contains supplementary material available at 10.1007/s00520-025-09225-y.

## Introduction

Almost one in five newly diagnosed cancer patients cares for a minor child [1]. Balancing the responsibilities of parenting minor children while battling cancer can exacerbate concerns not only about one’s own illness, but also about the well-being of the children [2]. Studies have shown conflicting results regarding the specific psychological effects for cancer patients parenting minor children [3]. Some studies reported higher depression, anxiety, and worry rates [4, 5], while others did not observe increased distress in cancer patients parenting minor children [6]. Nonetheless, cancer and parenthood pose unique challenges for patients, resulting in practical and emotional problems as well as a greater need for psychological support [6]. In its entirety, the psychological constructs of anxiety, depression, distress and worry are collectively regarded as *psychological burden* in this article.

Parental cancer affects not only the patient but the entire family, and the prevalence of mental disorders is heightened among all family members [7, 8]. Therefore, cancer is often referred to as a “we-disease” [9]. Partners play a major role in helping patients to deal with cancer and to cope with the resulting challenges and difficulties [10, 11]. Having a partner, compared with being single, is associated with positive outcomes such as higher quality of life and lower anxiety in cancer patients [11, 12]. Therefore, single motherhood compared to partnered motherhood may also be associated with reduced psychological well-being. In Germany about 19.9% of households with minor children are headed by single parents [13], resulting in approximately 5000 newly diagnosed single parents affected by cancer and caring for minor children each year (calculated based on total new diagnoses in Germany [14]). While the special needs of families with parental cancer have been acknowledged in recent years with an increase in specific interventions for families, less attention is paid to single parents with cancer [15]. Single parents with cancer often have to cope with their worries and fears alone, while caring for their children and managing daily life, and may therefore face increased difficulties.

The majority of single parents are mothers, representing 84.8% (Germany 2022) [13]. Studies on single mothers (SMs) revealed higher psychosocial and health risks and lower quality of life compared with partnered mothers (PMs) [16–19]. As patients with cancer have to confront even greater physical and psychological challenges, this vulnerability might be even higher for SMs affected by cancer. In a multi-case study of SMs with cancer, Behar [20] identified strong worries about dying, particularly in regard to the well-being and future of their children. These increased parental concerns might have further negative implications [21], as parental concerns have been associated with anxiety, depression, distress, and lower quality of life in cancer patients [8, 22]. Conversely, Elmberger, Bolund [23] reported in their qualitative multi-case study, that many SMs perceived newly developed strengths due to the life changes associated with their cancer, e.g., they valued life in a new way. To the authors knowledge, no quantitative study has investigated the extent of psychological burden and quality of life in the subgroup of SMs affected by cancer. Research is needed to determine whether single motherhood to minor children poses a risk factor for increased psychological problems in cancer patients, in order to identify and specifically support highly burdened patients in a timely manner. Therefore, the aims of this study were to (1) examine whether psychological burden (anxiety, depression, distress) and quality of life differ between SMs and PMs affected by cancer; and (2) identify potential group differences between SMs and PMs with cancer regarding reported practical, family, emotional, spiritual/religious, and physical problems, and parental concerns.

## Methods

### Design and data collection

The present secondary cross-sectional analysis was conducted using baseline data from the Family-SCOUT (F-SCOUT) study, a multicenter, quasi-experimental, non-randomized controlled trial of the effectiveness of a comprehensive support program for families with parental cancer and minor children. Sociodemographic as well as psychological measures were collected at baseline (T0) and at follow-up measurement points. The study protocol of the F-SCOUT study is described by Dohmen, Petermann-Meyer [24], background information and the primary analysis is given by Petermann-Meyer, Dohmen [25, 26]. Members of the public, healthcare providers, and patients participated in planning and developing F-SCOUT. F-SCOUT was registered in the Clinical Trial register (NCT04186923).

The present study is reported in accordance with the Strengthening the Reporting of Observational Studies in Epidemiology (STROBE) Statement on cross-sectional studies.

### Study setting and sampling

Recruitment of the primary F-SCOUT study was conducted at three sites of the German *Center of Integrated Oncology Aachen-Bonn-Cologne-Düsseldorf* (*CIO*^*ABCD*^) namely Aachen, Bonn, and Düsseldorf between October 2018 and December 2020 (partly during the COVID-19 pandemic). Participants were identified by multiprofessional oncology care teams and outreach partners of the *CIO*^*ABCD*^ cancer centers, who forwarded the participants’ contact information to the study sites in order to reduce bias due to access barriers.

### Inclusion and exclusion criteria

Eligibility criteria were as follows (1) families with at least one parent with cancer, or single parents with cancer (ICD-10 C diagnosis); (2) custody of at least one minor child or minor child living in the household (minor children were defined as children up to the age of 18); and (3) sufficient German language skills. Participants were excluded if: (1) they withdrew consent; (2) they had relevant cognitive impairments, preventing informed study consent. For the present secondary analysis, psychological measures at baseline (T0) were examined in the subgroup of women affected by cancer. Men were excluded from this analysis due to the small case numbers (*n* = 5 single fathers; *n* = 91 partnered fathers). Participants were divided into *SMs affected by cancer* and *PMs affected by cancer* on the basis of the sociodemographic question “Do you live with a partner?”. This classification was based on the definition of SMs provided by the German Federal Statistical Office, which states that: “Single parents are mothers and fathers who live together in a household with underage or adult children without a spouse or partner.” (translated from German [27]).

### Study variables

Psychological burden comprised anxiety, depression, and distress. Data analyzed in this study included the following variables using self-assessment questionnaires:

#### Sociodemographic and clinical characteristics

Sex, birth year, living with a partner (grouping variable, see 2.4), marital status, highest educational level, number and age of the children, currently being on sick leave, number of sick days during the last 3 months, employment, time since diagnosis, and type of cancer diagnosis and comorbidities were assessed.

#### Anxiety and depression

The German version of the Hospital Anxiety and Depression Scale (HADS [28]; German version [29]) was used. Participants’ anxiety and depression during the past week were measured at baseline (T0). Anxiety (HADS-A) and depression (HADS-D) are each measured with seven items on a four-point Likert scale ranging from 0 to 3. The reliability and validity of the HADS scale have been confirmed and extended to cancer patients [29, 30]. As recommended, cut-off scores of ≥ 8 were employed for the subscales [31] and scores between 8 and 10 were interpreted as mild, between 11 and 14 as moderate and between 15 and 21 as severe anxiety or depression [29]. For the total score, a cut-off score between 13 and 18 is reported as appropriate for various cancer samples [32]. For a conservative approach, the cut-off score was set at ≥ 16. In this study, the Cronbach’s $\alpha$ was 0.89.

#### Distress

Distress was assessed with the German version of the National Comprehensive Cancer Network (NCCN) Distress-Thermometer [33]. The Distress-Thermometer measures distress on a single-item visual analog scale ranging from 0 = *no distress* to 10 = *maximum distress*. In order to adequately balance between sensitivity and specificity, a cut-off score of ≥ 5 was used to indicate heightened distress [33]. Good internal consistency of the NCCN Distress-Thermometer can be assumed (*r* = 0.80 [33]), but validation studies suggest a two-step approach for diagnostic purposes [34].

#### Quality of life

Quality of life was measured with the German version of the European Quality of Life-5 Dimensions-5 Level Version visual analog scale (EQ-5D-5L [35]; German version [36]). Participants rated their current health on a 0 = *worst imaginable health state* to 100 = *best imaginable health state* vertical visual analog scale to assess their quality of life irrespective of cancer. The EQ-5D-5L visual analog scale has been validated and psychometric properties have been reported *(r* = 0.71–0.87 [37]).

#### Specific problems

Participants were asked about their specific problems using the German version of the NCCN problem list [33]. Patients report whether they experience problems in the practical (5 items, e.g. transportation, child care), family (2 items, e.g. dealing with partner), emotional (6 items, e.g. worry, depression), spiritual/religious (2 items, e.g. in relation to God) and physical context (21 items, e.g. pain, sleep). The NCCN problem list was specifically developed to assess common problems related to the cancer experience of oncological patients [38]. The exact questionnaire version used in this study can be found at Mehnert, Müller [33].

#### Parental concerns

Parental concerns were assessed using the German version of the Parenting Concerns Questionnaire (PCQ [15, 21]). The PCQ has been specifically developed to measure the severity of parental concerns among adults with cancer [21]. Participants rate their concerns using 15 items with five-point Likert scales ranging from 1 to 5. The instrument consists of three subscales: (1) practical impact of illness on child (e.g. the disease changing the child’s daily life), (2) emotional impact of illness on child (e.g. the child being emotionally agitated), and (3) concerns about co-parent (e.g. emotional and practical support by the co-parent; others caring for child in case of own death). A higher total score indicates more parental concerns. Within the questionnaire, it is assessed whether participants currently have a partner and whether the co-parent has passed away. For subscale 3, two items (12 and 13) did not apply to participants without a current partner (excluded items: “My partner is not providing me with enough emotional support”; “My partner is not providing me with enough practical support”). To participants with a deceased co-parent, two items (14 and 15) did not apply (excluded items: “My children’s other parent would not be able to meet their emotional needs if I die”; “My children’s other parent would not be a responsible caregiver if I die”). Good internal consistency and validity of the PCQ have been demonstrated ($\alpha$ = 0.93 [15]). The Cronbach’s $\alpha$ in this study was 0.88.

### Data analysis

Statistical analyses were performed using R Statistical Software (Version 4.3.1 [39]). Data cleaning and validation procedures were applied to identify and rectify any errors or inconsistencies in the dataset. A *p*-value of < 0.05 was considered statistically significant. Descriptive statistics were presented using frequencies and percentages for categorical data, continuous data were described with means (*M*), medians and standard deviations (*SD*). For the purposes of data analysis in this study, the age of children was categorized as under or over four years of age, as parenting young children is particularly labor intensive. This was further accentuated during the COVID-19 pandemic [40, 41]. For the questionnaires HADS, NCCN Distress Thermometer and Problem list as well as the EQ-5L-5D, reported scores correspond to sum scores. For the PCQ, reported scores correspond to sum scores divided by the number of items in order to correct for the different item numbers in the groups. To investigate potential bias due to differential sociodemographic and clinical characteristics between the participant groups, *t*-tests, Chi-squared tests (with Yates correction), Fisher’s exact tests and asymptotic two-sample Brown-Mood median tests were conducted to compare the groups. For the main analysis, missing variables were excluded and relevant distributional assumptions were checked. Outliers with an absolute *z*-score value greater than 3.29 were removed, as this cut-off value is a conventional criteria for identifying significant outliers [42]. To examine whether SMs and PMs differed regarding psychological burden and quality of life (aim 1) and to investigate specific problems and parental concerns of SMs (aim 2), *t*-tests for independent samples were calculated, evaluating group differences between SMs and PMs in the outcome variables anxiety and depression, total HADS score, distress, quality of life, current problems, and parental concerns. For the HADS questionnaire and the NCCN Distress-Thermometer, associations between symptom severity and partnership status were tested using Chi-squared tests.

#### Exploratory analyses

In order to control for sociodemographic and clinical differences potentially influencing our results, logistic regression models were conducted for outcomes with significant group differences in a second step. Therefore, we predicted the odds of belonging to the SMs group, while controlling for variables with significant differences between the groups (see Sect. 3.5). Seen as both groups reported mean ages within the same age group, age was not included as a predictor. Outliers with an absolute *z*-score value greater than 3.29 were also excluded from the logistic regression models.

## Results

### Participants

**Fig. 1:**
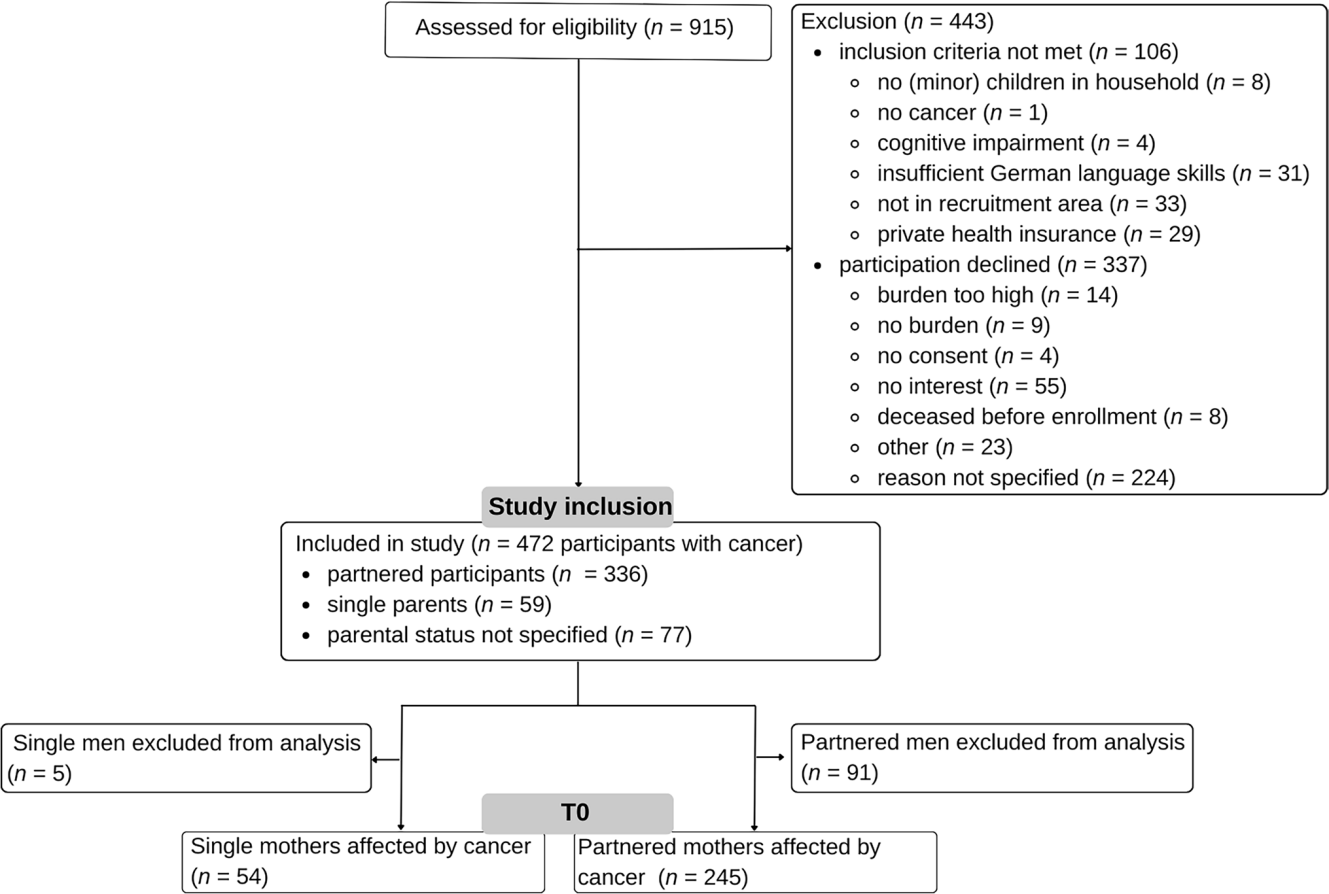
CONSORT flow diagram

Out of 915 persons assessed for eligibility for the F-SCOUT study, 11.6% were excluded due to unmet inclusion criteria and 36.8% declined participation (Fig. 1). In the current secondary analysis, *N* = 299 mothers affected by cancer were included (*n* = 54 SMs; *n* = 245 PMs). As completion rates varied for the different outcome variables, specific participant numbers and missing data are indicated for the following analyses.

### Characteristics of the sample

Most participants (82%) were living with a partner (PMs), while 18% were SMs (see Table 1). The proportion of SMs in this study was similar to the proportion of SMs in the German population (19.8% [43]). The age range was 22–61 years (SMs: 22–60; PMs: 23–61). The most common diagnosis in both groups was breast cancer (*n* = 158, 52.8%). SMs and PMs differed significantly in age (SMs > PMs), marital status (married: SMs < PMs; single, divorced, widowed: SMs > PMs), number of children under the age of four (SMs < PMs), currently being on sick leave (SMs > PMs), employment status (employed: SMs > PMs), and time since diagnosis (SMs < PMs). Detailed sociodemographic and clinical charateristics of participants can additionally be found in the supplementary information Table 1A.

**Table 1:** Sociodemographic and clinical characteristics of participants

Characteristic	Total *N* = 299		Single mothers *n* = 54 (18.1%)		Partnered mothers *n* = 245 (81.9%)			*p*
	*n* (m)	%	*n* (m)	%	*n* (m)	%		
*Age*	299 (0)	100 (0)	54 (0)	100 (0)	245 (0)		100 (0)	
Mean; *SD*	42.6	7.0	44.8	6.5	42.1		7.0	**0.010**^**a**^
*Marital status*	296 (3)	99 (1)	51 (3)	94 (6)	245 (0)		100 (0)	** > 0.001**^b^
Married	230	78	7	14	223		91	** > 0.001**^b^
Widowed	6	2	6	12	0		0	** > 0.001**^b^
Single	34	12	17	33	17		7	** > 0.001**^b^
Divorced	26	9	21	41	5		2	** > 0.001**^b^
*Highest educational level (classified)*	298 (1)	100 (0)	54 (0)	100 (0)	244 (1)		100 (0)	0.113^c^
< 12 years	93	31	20	38	73		30	
≥ 12 years	199	67	33	61	166		68	
Other	6	2	1	2	5		2	
*Child *<* 4 years*	297 (2)	99 (1)	54 (0)	100 (0)	243 (2)		99 (1)	**0.002**^b^
Yes	78	26	5	9	73		30	
No	219	74	49	91	170		70	
*Currently on sick leave*	278 (21)	93 (7)	52 (2)	96 (4)	226 (19)		92 (8)	**0.034**^c^
Yes	170	61	39	75	131		58	
No	108	39	13	25	95		42	
*Number of sick days last 3 months*	205 (94)	69 (31)	38 (16)	70 (30)	167 (78)		68 (32)	
Mean; *SD*	33.6 (21)	± 29.4	35.3 (30)	± 25.6	33.2 (20)		± 30.3	0.661^a^
Employment	278 (21)	93 (7)	50 (4)	93 (7)	228 (17)		93 (7)	**0.011**^b^
Employed	206	74	42	84	164		72	
Unemployed	72	26	8	16	64		28	
*Time since diagnosis (months)*	280 (19)	94 (6)	47 (7)	87 (13)	233 (12)		95 (5)	
Mean (median); *SD*	24.9 (6)	46.12	17.2 (6)	22.8	26.5 (6)		49.4	**0.047**^a^ (0.916^d^)
< 3 months	100	33	16	30	84		34	0.794^a^
Diagnosis*	299 (0)	100 (0)	54 (0)	100 (0)	245 (0)		100 (0)	
Breast cancer	158	53	30	56	125		51	0.650^c^
Leukemia	17	6	3	6	13		5	1^b^
Gastro-intestinal cancer (colorectal, gastric, liver, peritoneum)	21	7	2	4	18		7	0.829^b^
Pancreatic cancer	5	2	1	2	4		2	1^b^
Thyroid cancer	3	1	2	4	1		0	0.451^b^
Bladder cancer	3	1	0	0	3		1	1^b^
Brain cancer	18	6	5	9	13		5	0.338^b^
Gynecological cancer (ovarian, cervical, vaginal, other)	32	11	4	7	28		11	0.864^b^
Skin cancer	15	5	2	4	13		5	1^b^
Lung cancer	7	2	2	4	5		2	0.614^b^
Osteosarcoma/bone cancer	5	2	1	3	4		2	1^b^
Lymphoma	17	6	2	4	14		6	0.745^b^
Other (e.g., laryngeal, sarcoma)	13	4	1	2	12		5	0.475^b^

*Notes: p*-values refer to the statistical tests performed for the comparison of mean values and frequencies of sociodemographic characteristics between single mothers with cancer and partnered mothers with cancer. Bold values indicate significant results (*p* < 0.05). Marital status refered to the current status, as in some cases SMs, e.g. lived without a partner, but remained married. Highest educational level < 12 years = none/basic school attendance (8–9 years), middle maturity/secondary (10 years); highest educational level ≥ 12 years = college degree/specialized A-levels (11–12 years), university entrance qualification/high school diploma/A-levels (12–13 years); employed = full-time, part-time > 50%, self-employed, part-time up to 50%, occupational rehabilitation; unemployed = unemployed, fully disabled, pensioner, housewife, pupil/student; m = missing data

* Multiple selections due to primary, secondary, tertiary cancer; percentages for cancer diagnoses were calculated based on case numbers

^a^ = calculated using *t*-test. ^b^ = calculated using Fisher’s exact test*.* ^c^ = calculated using Chi-squared test with Yates correction. ^d^ = calculated using asymptotic two-sample Brown-Mood median test

### Description of psychological outcomes in the sample

A detailed overview over means and standard deviations of all outcomes is provided in the supplementary files (Table 2A).

**Table 2:** Logistic regression models predicting single motherhood with predictor practical problems controlling for sociodemographic and clinical variables

Predictor	Estimate	*Z*	*p*	Odds ratio	95%-CI
*Model 1*					
Practical problems	0.378	2.611	**0.009**	1.460	[1.10; 1.95]
*Model 2*					
Practical problems	0.461	3.100	**0.002**	1.586	[1.19; 2.14]
Child < 4 years	− 2.030	− 3.257	**0.001**	0.131	[0.03; 0.38]
*Model 3*					
Practical problems	0.419	2.827	**0.005**	1.520	[1.14; 2.04]
Employment status	0.786	1.920	0.055	2.193	[1.02; 5.17]
*Model 4*					
Practical problems	0.341	2.270	**0.023**	1.407	[1.05; 1.89]
Time since diagnosis	− 0.005	− 1.013	0.311	0.995	[0.98; 1.00]
*Model 5*					
Practical problems	0.356	2.412	**0.016**	1.428	[1.07; 1.91]
Currently on sick leave	− 0.666	− 1.795	0.073	0.514	[0.24; 1.04]
*Model 6*					
Practical problems	0.425	2.625	**0.009**	1.530	[1.12; 2.12]
Child < 4 years	− 1.937	− 3.063	**0.002**	0.144	[0.03; 0.43]
Employment status	0.390	0.784	0.433	1.477	[0.58; 4.12]
Time since diagnosis	− 0.007	− 1.349	0.177	0.993	[0.98; 1.00]
Currently on sick leave	− 0.471	− 1.083	0.279	0.624	[0.26; 1.43]

*Notes.* Time since diagnosis measured in months. Bold values indicate significant results (*p* < 0.05). Outliers were removed (*z* > 3.29); practical problems: 2 outliers removed

SMs and PMs both indicated clinically relevant HADS values with 176 participants (58.9%) presenting total HADS scores above the cut-off (≥ 16), 59.3% in the SMs group and 58.8% in the PMs group. With regard to anxiety, 71.9% participants (SMs: 64.8%; PMs: 73.5%) were identified above the cut-off, with 27.8% reporting mild (SMs: 18.5%; PMs: 29.8%), 29.4% moderate (SMs: 35.2%; PMs: 28.2%) and 14.7% severe anxiety (SMs: 11.1%; PMs: 15.5%). Clinical levels of depression were identified in 46.8% participants (SMs: 48.1%; PMs: 46.5%), 23.1% reported mild (SMs: 18.5%; PMs: 24.5%), 13.4% moderate (SMs: 16.7%; PMs: 12.7%) and 10% severe depression (SMs: 13%; PMs: 9.4%).

Of the participants, 82.3% reported moderate distress with scores above the cut-off (≥ 5): 81.5% participants in the SMs group and 82.8% in the PMs group.

### Analyses of differences in psychological outcomes between SMs and PMs

*T*-tests for independent samples showed no significant differences between the SMs and PMs for the HADS total score (*t*(295) = − 0.74, *p* = 0.463), anxiety (*t*(295) = − 0.87, *p* = 0.384), depression (*t*(296) = − 0.27, *p* = 0.786), distress (*t*(280) = − 0.32, *p* = 0.746; one outlier removed), and quality of life (*t*(295) = 1.18, *p* = 0.240). Furthermore, there were no differences in emotional (*t*(257) = 1.94, *p* = 0.054), family (*t*(270) = − 1.09, *p* = 0.278), spiritual/religious (*t*(282) = − 0.30, *p* = 0.766; 16 outliers removed), and physical problems (*t*(242) = − 0.29, *p* = 0.774) between SMs and PMs.

However, SMs reported significantly more practical problems (*t*(266) = 2.68, *p* = 0.008, *d* = 0.439 [0.115; 0.763]; two outliers removed). Parental concerns differed between SMs and PMs (*t*(270) = − 2.55, *p* = 0.011, *d* = 0.399 [0.089; 0.709]), specifically on the “concerns about co-parent” subscale (*t*(283) = − 5.64, *p* < 0.001, *d* = 0.872 [0.559; 1.185]), but not on the “practical impact” (*t*(291) = 0.61, *p* = 0.544) and “emotional impact” (*t*(289) = − 0.95, *p* = 0.342) subscales. Not all items on the “concerns about co-parent” scale were relevant for all participants, as some participants had no partner or a deceased co-parent (see 2.4.). Specifically, 29 SMs had no partner and 22 participants reported that their co-parent had died (13 SMs, 9 PMs).

**Fig. 2:**
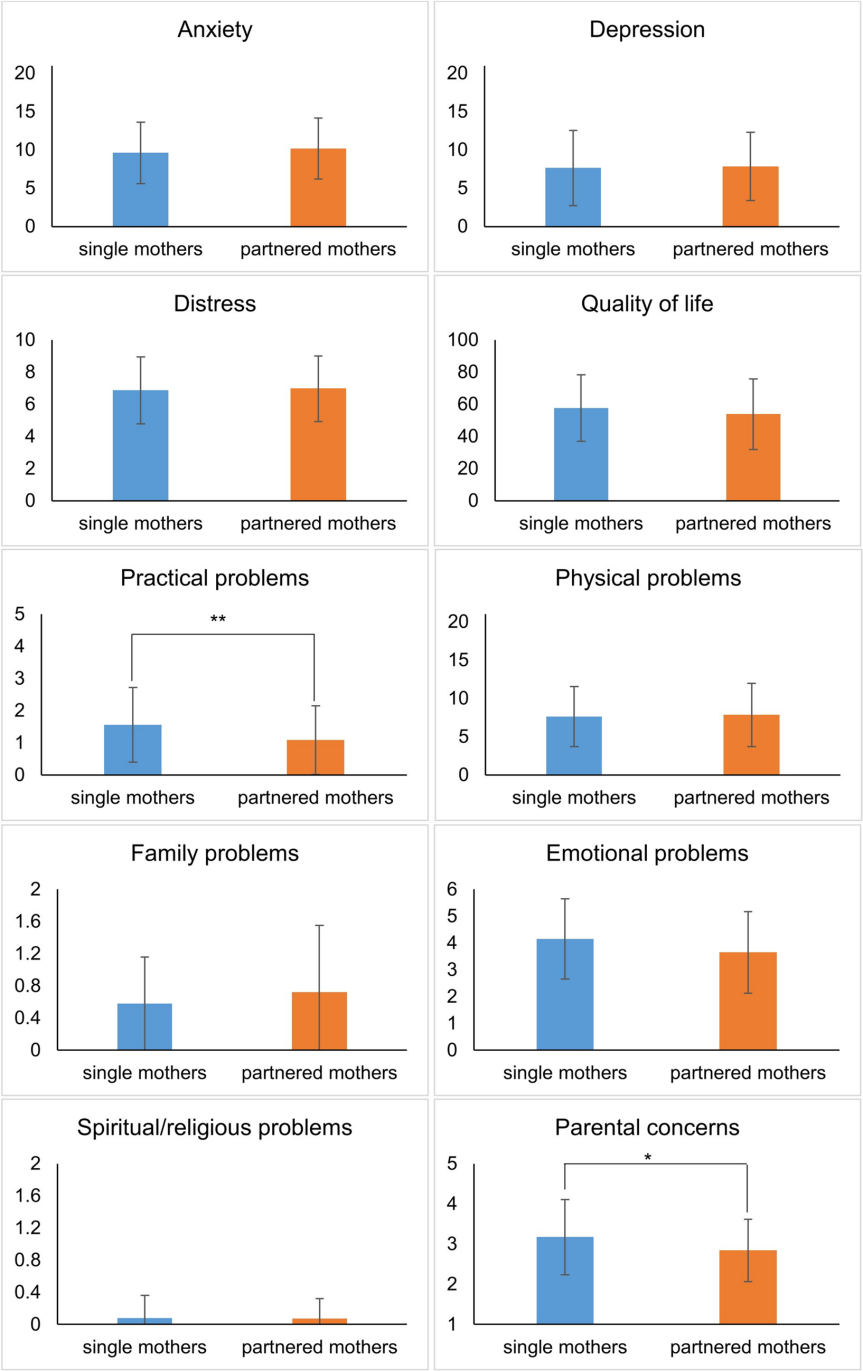
Means and standard deviations of all outcomes for single and partnered mothers affected by cancer. *Notes.* Statistical calculations were performed using *t*-tests. Questionnaires: Hospital Anxiety and Depression Scale for anxiety and depression; National Comprehensive Cancer Network Distress Thermometer for distress; European Quality of Life—5 Dimensions—5 Level Version for quality of life; National Comprehensive Cancer Network Problem List for practical, physical, family, emotional, and spiritiual/religious problems; Parenting Concerns Questionnaire for parental concerns. Values for parental concerns correspond to sum scores divided by number of items, all other values correspond to sum scores.* *p* < 0.05; ** *p* < 0.01

There was no association between partnership status and heightened distress, anxiety severity, or depression severity (NCCN Distress-Thermometer: χ2(1) = 0.00, p = 1, HADS-A: χ2(3) = 4.22, p = 0.239; HADS-D: χ2(3) = 1.78, p = 0.620). For a detailed overview, see Fig. 2.

### Exploratory analyses

Logistic regression models predicting single motherhood with the predictors practical problems, child under four years, employment status, time since diagnosis, and current sick leave confirmed a significant association of practical problems with parental status (Table 2). After controlling for sociodemographic and clinical variables that differed between the two groups, an increase of one practical problem was associated with a 53% increase in the odds of belonging to the SMs group.

Similarly, after controlling for child under four years, employment status, time since diagnosis, and current sick leave, parental concerns remained significantly associated with single motherhood. An increase of one parental concern was associated with a 73% increase in the odds of belonging to the SMs group (see Table 3).

**Table 3:** Logistic regression models predicting single motherhood with predictor parental concerns controlling for sociodemographic and clinical variables

Predictor	Estimate	*Z*	*p*	Odds ratio	95%-CI
*Model 1*					
PCQ	0.493	2.495	**0.013**	1.637	[1.12; 2.43]
*Model 2*					
PCQ	0.458	2.328	**0.020**	1.580	[1.08; 2.34]
Child under 4 years	− 1.590	− 2.927	**0.003**	0.204	[0.06; 0.53]
*Model 3*					
PCQ	0.586	2.808	**0.005**	1.796	[1.20; 2.73]
Employment status	0.941	2.298	**0.022**	2.56	[1.20; 6.05]
*Model 4*					
PCQ	0.482	2.294	**0.022**	1.619	[1.08; 2.47]
Time since diagnosis (months)	− 0.005	− 1.00	0.317	0.995	[0.98; 1.00]
*Model 5*					
PCQ	0.523	2.522	**0.012**	1.688	[1.13; 2.56]
Currently on sick leave	− 0.753	− 2.092	**0.036**	0.471	[0.23; 0.93]
*Model 6*					
PCQ	0.545	2.421	**0.015**	1.725	[1.12; 2.72]
Child under 4 years	− 1.811	− 2.901	**0.004**	0.163	[0.04; 0.48]
Employment status	0.374	0.739	0.460	1.454	[0.56; 4.12]
Time since diagnosis	− 0.006	− 1.076	0.282	0.994	[0.98; 1.00]
Currently on sick leave	− 0.541	− 1.272	0.203	0.582	[0.24; 1.31]

*Notes. PCQ*, Parenting Concerns Questionnaire. Bold values indicate significant results (*p *< 0.05). Time since diagnosis measured in months

## Discussion

The primary aim of this study was to investigate whether psychological burden and quality of life differ between SMs and PMs. The secondary aim was to identify the specific problems SMs with cancer may face as well as potential differences in parental concerns. While no significant group differences were observed for the primary aim, more practical problems and parental concerns were reported in SMs than in PMs.

Overall, the results show that anxiety, depression, and distress are frequently prevalent among both SMs and PMs. The quality of life of all participants was observed to be relatively low in comparison to the normative data for the German population [44]. Heightened distress was reported by 82.3% of the sample, and anxiety and depression above the cut-off were reported by 71.9% and 46.8% of participants, respectively.

Concerning the primary aim, no significant group differences were observed for psychological burden (anxiety, depression, distress) or quality of life between SMs and PMs. These findings seem surprising in light of previous literature, reporting that SMs experience greater distress and are more likely to suffer from mental disorders than married mothers [16, 17]. Studies in cancer patients outlined the supportive role of partners associated with lower distress [10, 11]. However, to our knowledge, no study has yet investigated the potential differences in psychological burden and quality of life of SMs and PMs including all cancer entities. Among women with breast cancer, having a partner was shown to predict lower anxiety and higher quality of life [11, 12]. This stands in contrast with our observations among mothers with different types of cancer. A possible explanation for this discrepancy with previous studies could be that different cancer entities pose different challenges to SMs and PMs. Therefore, the findings on distress can presumably not be generalized from one cancer entity to other cancer entities and motherhood could add additional challenges. However, given the high prevalence of individuals reporting psychological burden in the current sample examined, a possible ceiling effect and a floor effect for quality of life cannot be excluded, as SMs and PMs with cancer are both confronted with an extremely stressful situation. Furthermore, global measures such as the HADS or the NCCN Distress-Thermometer might insufficiently identify the specific problems and challenges faced by SMs with cancer. The results could also hint at high coping strategies of SMs in this sample, who might have found flexible strategies in dealing with their difficult situation. Overall, a high number of mothers in both groups–SMs and PMs affected by cancer–experienced acute psychological burden and reduced quality of life. In addition, the high prevalence of anxiety and depression may have detrimental health consequences, as associations between mental disorders and morbidity as well as mortality have been shown [45]. However, based on the results of this study, SMs and PMs with cancer do not appear to differ regarding their psychological burden and quality of life. Future investigations are needed to address a potential ceiling and floor effect as well as to identify the specific impact of different cancer entities when comparing the psychological burden and quality of life in SMs and PMs.

Concerning the secondary aim, we observed that SMs with cancer reported more practical problems in their daily life (e.g., insurance, transport, childcare) compared with PMs. Furthermore, practical problems were associated with single motherhood, even when controlling for relevant sociodemographic and clinical characteristics such as children under the age of four, and time since diagnosis. Despite previous findings of lower health among SMs and an important role of the partner regarding emotional support [11, 46], physical and emotional problems did not differ between SMs and PMs with cancer. As Fugmann, Richter [6] have shown more practical and emotional problems in cancer patients with minor children compared with patients without minor children, the current study extends the scientific knowledge to the subgroup of SMs affected by cancer. These findings indicate more obstacles in daily life for SMs than for PMs with cancer, and that living with a partner might relieve practical problems.

Moreover, and in line with preliminary findings from a multi-case study, SMs with cancer reported greater parental concerns compared with PMs with cancer [20]. Additionally, after controlling for sociodemographic and clinical characteristics, more parental concerns remained associated with higher odds of belonging to the SM group. Therefore, we conclude that the reported differences in parental concerns between SMs and PMs cannot be explained solely by group differences in these sociodemographic and clinical characteristics. Behar [20] identified strong worries in SMs affected by cancer about their children’s well-being and future in the context of fear about their own death. These results are consistent with our findings, as the differences in parental concerns between SMs and PMs in our study were driven by the subscale focusing primarily on partner support and caring for children after one’s own death. Since increased parental concerns were associated with anxiety, depression, and a lower quality of life in cancer patients in a previous study [22], future studies should aim to identify potential characteristics of SMs that are especially prone to parental concerns, as they might lead to detrimental psychological well-being in the future.

### Limitations

The main limitation of the present study is its cross-sectional design, which excludes the possibility of employing multivariate correlational and longitudinal analyses. Consequently, the conclusions that can be drawn are restricted to a descriptive analysis of the studied population.

In addition, there may be a selection bias in the recruitment of participants, as despite the active outreach approach, the main study included predominantly highly stressed families. This bias could have resulted in a limited range of scores, explaining the high burden in both SMs and PMs. In addition, part of the recruitment took place during the COVID-19 pandemic, which may have further affected families due to restrictions on hospital visits. Another limitation is that parental status was assessed as a categorical variable based on the definition from the German Federal Statistical office (living with a partner: yes/no), not taking into account the relationship duration and quality. The support a partner provided could have differed based on a multitude of factors, potentially influencing the reported results. In addition, a small percentage of SMs reported that they currently had a partner, who however did not live in their home. In addition, the items assessed for parental concerns differed between participants, reflecting their different co-parenting and partnership situations. However, this may have influenced our results as a higher number of SMs completed a smaller number of items than participants in the PM group. Additionally, the diversity of the population in terms of cancer entities is a limitation, as they are associated with different stressors and limitations in daily life.

### Recommendations for further research

Future studies should investigate psychological outcomes of mothers affected by cancer using a longitudinal design in order to investigate the persistence of psychological burden, reported problems, and parental concerns. Additionally, research should test the impact of cancer entities as potential moderators for the relationship between partnership status and psychological outcomes. As partnership and co-parenting status of mothers also directly influences their children, the potential impact of partnership status on the psychological burden of children should also be investigated. Furthermore, as welfare systems and national healthcare differ substantially between countries, international comparative studies are needed to examine the transferability of results. Lastly, to extend knowledge to the specific problems and concerns of single parents in general, single fathers should also be addressed in further research.

### Clinical implications

These results highlight the necessity of clinical support for PMs and SMs affected by cancer, as both groups reported high prevalence of psychological burden and a reduced quality of life. Furthermore, the results provide additional insights into the specific situation of SMs and can steer healthcare professionals to improve identification of stressors faced by SMs. As the specific problems and concerns of SMs could not be identified using global measures, distinct measurement tools should be implemented in order to appropriately identify individuals with high support needs. In a second step, support services can be tailored to the specific situation of SMs accordingly, as this study identified the relevance of additional social support in the areas of practical problem solving, parental concerns, and psychosocial care in addition to dealing with psychological burden. Specifically, social services should prioritize assisting SMs with cancer in areas such as housing, childcare, transportation, work/school, and insurance. For instance, offering childcare during intensive cancer treatment could provide significant relief. Furthermore, given the identified concerns about ensuring adequate care for children after the one’s own death, interventions should include the development of preventative plans with patients, addressing end-of-life care arrangements for their children.

## Conclusion

SMs affected by cancer who are caring for minor children did not report higher psychological burden or lower quality of life than PMs. However, they experienced more practical problems and parental concerns. As these group differences were not fully explained by sociodemographic or clinical factors, single motherhood itself seems to play a significant role. Furthermore, both SMs and PMs showed high prevalence of psychological burden and reduced quality of life, highlighting the challenging situation of mothers with cancer. These results may help to better understand the psychological challenges mothers with cancer, especially SMs, face and can provide further guidance for tailored clinical support services.

## Supplementary Information

Below is the link to the electronic supplementary material.

Supplementary file1 (DOCX 24 KB)

## Data Availability

The data that support the findings of this study are available on request from the corresponding author. The data are not publicly available due to privacy or ethical restrictions.
