# Supplementary material for: Psychological burden, quality of life, problems, and parental concerns of single mothers with cancer: a cross-sectional comparison
Source: Support Care Cancer. 2025 Feb 11;33(3):173. doi: 10.1007/s00520-025-09225-y (PMC11814052; doi:10.1007/s00520-025-09225-y)
Supplement: Supplementary file 1 — Supplementary file1 (DOCX 24 KB) [file 520_2025_9225_MOESM1_ESM.docx]

**SUPPLEMENTARY INFORMATION**

**Table 1A**

*Sociodemographic and clinical characteristics of participants*

| **Characteristic** | **Total**  *N* = 299 | |  | **Single Mothers**  *n* = 54 (18.1%) | |  | **Partnered Mothers**  *n* = 245 (81.9%) | | | ***p*** |
| --- | --- | --- | --- | --- | --- | --- | --- | --- | --- | --- |
|  | ***n* (m)** | **%** |  | ***n* (m)** | **%** |  | ***n* (m)** | ***%*** | |  |
| **Age** | 299 (0) | 100 (0) |  | 54 (0) | 100 (0) |  | 245 (0) | | 100 (0) |  |
| Mean; *SD* | 42.6 | 7.0 |  | 44.8 | 6.5 |  | 42.1 | | 7.0 | **.010^a^** |
| **Marital status** | 296 (3) | 99 (1) |  | 51 (3) | 94 (6) |  | 245 (0) | | 100 (0) | **> .001**^b^ |
| Married | 230 | 78 |  | 7 | 14 |  | 223 | | 91 | **> .001**^b^ |
| Widowed | 6 | 2 |  | 6 | 12 |  | 0 | | 0 | **> .001**^b^ |
| Single | 34 | 12 |  | 17 | 33 |  | 17 | | 7 | **> .001**^b^ |
| Divorced | 26 | 9 |  | 21 | 41 |  | 5 | | 2 | **> .001**^b^ |
| **Highest educational level (classified)** | 298 (1) | 100 (0) |  | 54 (0) | 100 (0) |  | 244 (1) | | 100 (0) | 0.113^c^ |
| None/basic school attendance (8-9 years) | 30 | 10 |  | 10 | 19 |  | 20 | | 8 |  |
| Middle maturity/Secondary (10 years) | 63 | 21 |  | 10 | 19 |  | 53 | | 22 |  |
| College degree/specialised A-levels (11-12 years) | 42 | 14 |  | 7 | 13 |  | 35 | | 14 |  |
| University entrance qualification/ high school diploma/A-levels (12-13 years) | 157 | 53 |  | 26 | 48 |  | 131 | | 54 |  |
| Other | 6 | 2 |  | 1 | 2 |  | 5 | | 2 |  |
| **Child < 4 Years** | 297 (2) | 99 (1) |  | 54 (0) | 100 (0) |  | 243 (2) | | 99 (1) | **.002**^b^ |
| Yes | 78 | 26 |  | 5 | 9 |  | 73 | | 30 |  |
| No | 219 | 74 |  | 49 | 91 |  | 170 | | 70 |  |
| **Currently on sick leave** | 278 (21) | 93 (7) |  | 52 (2) | 96 (4) |  | 226 (19) | | 92 (8) | **.034**^c^ |
| Yes | 170 | 61 |  | 39 | 75 |  | 131 | | 58 |  |
| No | 108 | 39 |  | 13 | 25 |  | 95 | | 42 |  |
| **Sick days last 3 months** | 288 (11) | 96 (4) |  | 53 (1) | 98 (2) |  | 235 (10) | | 96 (4) |  |
| Yes | 226 | 79 |  | 41 | 77 |  | 185 | | 79 | .832^a^ |
| **Number of sick days last 3 months** | 205 (94) | 69 (31) |  | 38 (16) | 70 (30) |  | 167 (78) | | 68 (32) |  |
| Mean; *SD* | 33.6 (21) | ± 29.4 |  | 35.3 (30) | ± 25.6 |  | 33.2 (20) | | ± 30.3 | .661^a^ |
| **Employment** | 278 (21) | 93 (7) |  | 50 (4) | 93 (7) |  | 228 (17) | | 93 (7) | **.011**^b^ |
| Full-time | 43 | 16 |  | 10 | 20 |  | 33 | | 15 | .446^c^ |
| Part-time > 50% | 64 | 23 |  | 14 | 28 |  | 50 | | 22 | .46^c^ |
| Self-employed | 13 | 5 |  | 2 | 4 |  | 11 | | 5 | 1^b^ |
| Part-time up to 50% | 83 | 30 |  | 15 | 30 |  | 68 | | 30 | 1^c^ |
| Occupational rehabilitation | 3 | 1 |  | 1 | 2 |  | 2 | | 1 | .450^b^ |
| Unemployed | 14 | 5 |  | 4 | 8 |  | 10 | | 4 | .289^b^ |
| Fully disabled | 12 | 4 |  | 1 | 2 |  | 11 | | 5 | .7^b^ |
| Pensioner | 5 | 2 |  | 2 | 4 |  | 3 | | 1 | .221^b^ |
| Housewife | 39 | 14 |  | 0 | 0 |  | 39 | | 17 | **>.001**^b^ |
| Pupil/ Student | 2 | 1 |  | 1 | 2 |  | 1 | | 0 | .328^b^ |
| **Time since diagnosis (months)** | 280 (19) | 94 (6) |  | 47 (7) | 87 (13) |  | 233 (12) | | 95 (5) |  |
| Mean (Median); *SD* | 24.9 (6) | 46.12 |  | 17.2 (6) | 22.8 |  | 26.5 (6) | | 49.4 | **.047**^a^  (.916^d^) |
| < 3 months | 100 | 33 |  | 16 | 30 |  | 84 | | 34 | .794^a^ |
| **Diagnosis *** | 299 (0) | 100 (0) |  | 54 (0) | 100 (0) |  | 245 (0) | | 100 (0) |  |
| Breast cancer | 158 | 53 |  | 30 | 56 |  | 125 | | 51 | .650^c^ |
| Leukemia | 17 | 6 |  | 3 | 6 |  | 13 | | 5 | 1^b^ |
| Gastro-intestinal cancer (colorectal, gastric, liver, peritoneum) | 21 | 7 |  | 2 | 4 |  | 18 | | 7 | .829^b^ |
| Pancreatic cancer | 5 | 2 |  | 1 | 2 |  | 4 | | 2 | 1^b^ |
| Thyroid cancer | 3 | 1 |  | 2 | 4 |  | 1 | | 0 | .451^b^ |
| Bladder cancer | 3 | 1 |  | 0 | 0 |  | 3 | | 1 | 1^b^ |
| Brain cancer | 18 | 6 |  | 5 | 9 |  | 13 | | 5 | .338^b^ |
| Gynecological cancer (ovarian, cervical, vaginal, other) | 32 | 11 |  | 4 | 7 |  | 28 | | 11 | .864^b^ |
| Skin cancer | 15 | 5 |  | 2 | 4 |  | 13 | | 5 | 1^b^ |
| Lung cancer | 7 | 2 |  | 2 | 4 |  | 5 | | 2 | .614^b^ |
| Osteosarcoma/Bone cancer | 5 | 2 |  | 1 | 3 |  | 4 | | 2 | 1^b^ |
| Lymphoma | 17 | 6 |  | 2 | 4 |  | 14 | | 6 | .745^b^ |
| Other (e.g. laryngeal, sarcoma) | 13 | 4 |  | 1 | 2 |  | 12 | | 5 | .475^b^ |
| **Treatment** *former and/or future* | 296 (3) | 99 (1) |  | 51(3) |  |  | 245 (0) | | 100 (0) |  |
| Surgery | 247 | 83 |  | 43 | 84 |  | 204 | | 83 | 1^c^ |
| Chemotherapy | 206 | 70 |  | 34 | 67 |  | 172 | | 70 | .740^c^ |
| Radiotherapy | 152 | 51 |  | 26 | 51 |  | 126 | | 51 | 1^c^ |
| Other | 111 | 38 |  | 23 | 45 |  | 88 | | 36 | .283^c^ |
| None | 1 | 0 |  | 0 | 0 |  | 1 | | 0 | 1^b^ |
| **Comorbidities** | 295 (4) | 99 (1) |  | 53 (1) | 98 (2) |  | 242 (3) | | 99 (1) |  |
| Physical | 127 | 43 |  | 21 | 40 |  | 106 | | 44 | .579^a^ |
| Mental | 45 | 15 |  | 12 | 23 |  | 33 | | 14 | 0.152^a^ |

*Notes: p*-values refer to the statistical tests performed for the comparison of mean values (age) and frequencies of sociodemographic characteristics between single mothers with cancer and partnered mothers with cancer. Marital status refered to the current status, as in some cases SMs e.g. lived without a partner, but remained married. m=missing data

*** Multiple selections due to primary, secondary, tertiary cancer; percentages for cancer diagnoses were calculated based on case numbers.

^a^ = calculated with *t*-test. ^b^ = calculated using Fisher’s exact test*.* ^c^ = calculated using Chi squared test with Yates correction. ^d^ = calculated using Asymptotic two-sample Brown-Mood median test.

**Table 2A**

*Number of participants, means and standard deviations of the outcome measures HADS, NCCN, EQ-5D-5L and PCQ*

| **Measure** | **Total**  *N* = 299 | | |  | **Single Mothers**  *n* = 54 | | |  | **Partnered mothers**  *n* = 245 | | |  | ***p*** | ***d*** |
| --- | --- | --- | --- | --- | --- | --- | --- | --- | --- | --- | --- | --- | --- | --- |
|  | ***n* (m)** | ***M*** | ***SD*** |  | ***n* (m)** | ***M*** | ***SD*** |  | ***n* (m)** | ***M*** | ***SD*** |  |  |  |
| **HADS Total** (range 0-42) | 297 (2) | 17.84 | 7.79 |  | 53 (1) | 17.13 | 8.38 |  | 244 (1) | 18.00 | 7.67 |  | .463 |  |
| HADS-Anxiety  (range 0-21) | 297 (2) | 10.08 | 3.98 |  | 53 (1) | 9.65 | 4.08 |  | 244 (1) | 10.17 | 3.96 |  | .384 |  |
| HADS-Depression  (range 0-21) | 298 (1) | 7.79 | 4.53 |  | 54 (0) | 7.64 | 4.90 |  | 244 (1) | 7.83 | 4.45 |  | .786 |  |
| **NCCN Distress-Thermometer** (range 0-10) | 282 (17) | 6.95 | 2.04 |  | 51 (3) | 6.86 | 2.07 |  | 231 (14) | 6.97 | 2.04 |  | .746 |  |
| **EQ-5D-5L visual analog scale** (range 0-100) | 297 (2) | 54.45 | 21.81 |  | 54 (0) | 57.61 | 20.64 |  | 243 (2) | 53.75 | 22.04 |  | .240 |  |
| **NCCN Problem List** | 210 (89) | 13.55 | 6.02 |  | 30 (24) | 13.07 | 5.31 |  | 180 (65) | 13.63 | 6.14 |  | .638 |  |
| Practical Problems*  (range 0-5) | 268 (29) | 1.16 | 1.09 |  | 45 (9) | 1.56 | 1.16 |  | 223 (20) | 1.08 | 1.07 |  | **.008** | 0.439 |
| Physical Problems  (range 0-21) | 244 (55) | 7.80 | 4.09 |  | 40 (14) | 7.63 | 3.93 |  | 204 (41) | 7.83 | 4.13 |  | .774 |  |
| Family problems  (range 0-2) | 272 (27) | 0.69 | 0.79 |  | 45 (9) | 0.58 | 0.58 |  | 227 (18) | 0.72 | 0.83 |  | .278 |  |
| Emotional Problems  (range 0-6) | 259 (40) | 3.73 | 1.52 |  | 41 (13) | 4.15 | 1.49 |  | 218 (27) | 3.65 | 1.52 |  | .054 |  |
| Spiritual/  Religious Problems* (range 0-2) | 268 (15) | 0.07 | 0.26 |  | 48 (4) | 0.08 | 0.28 |  | 220 (11) | 0.07 | 0.25 |  | .712 |  |
| **PCQ** (range 1-5) | 272 (27) | 2.90 | 0.81 |  | 50 (4) | 3.17 | 0.94 |  | 222 (23) | 2.84 | 0.78 |  | **.011** | 0.399 |
| Practical impact | 293  (6) | 3.32 | 0.96 |  | 54 (0) | 3.25 | 1.04 |  | 239 (6) | 3.34 | 0.94 |  | .544 |  |
| Emotional impact | 291 (8) | 3.13 | 1.00 |  | 53 (1) | 3.25 | 1.02 |  | 238 (7) | 3.11 | 1.00 |  | .342 |  |
| Concerns co-parent | 285 (14) | 2.29 | 1.12 |  | 51 (3) | 3.05 | 1.38 |  | 234 (11) | 2.12 | 0.99 |  | **< .001** | 0.872 |

*Notes.* Measures for single mothers with cancer and partnered mothers with cancer; statistical calculations performed using *t-*tests. Range refers to the possible minimum and maximum values and not to the range within the participants.

m=missing data; HADS = Hospital Anxiety and Depression Scale; NCCN = National Comprehensive Cancer Network Distress-Thermometer; EQ-5D-5L = European Quality of Life - 5 Dimensions - 5 Level Version; PCQ = Parenting Concerns Questionnaire; *d* = Cohen´s *d.*

* Outliers were removed (z > 3.29); practical problems: 2 outliers for partnered mothers; spiritual/religious problems: 2 outliers for single mothers, 14 outliers for partnered mothers
